# Successful Management of Severe and Refractory Autoimmune Hemolytic Anemia (AIHA) in a Sickle Cell Disease Patient With Bortezomib

**DOI:** 10.7759/cureus.74840

**Published:** 2024-11-30

**Authors:** Mohammed A Alanssari, Elaf Al-Sulaitti, Zainab Al-Sulaitti, Asaad A Khalaf, Qutaiba M Dawood

**Affiliations:** 1 Hematology, Basrah Health Directorate, Basrah, IRQ; 2 Hematology, Basrah Teaching Hospital, Basrah, IRQ; 3 Accident and Emergency, University Hospital Hairmyres, Glasgow, GBR; 4 Hematology and Medical Oncology, Al-Zahraa Medical College, Basrah, IRQ

**Keywords:** bortezomib, hemolysis, proteasome inhibitors, sickle cell anemia, w-aiha, warm antibody hemolytic anemia

## Abstract

Autoimmune hemolytic anemia (AIHA) is a multifactorial disease that causes immune-mediated red blood cell destruction, resulting in anemia and hemolysis symptoms. Despite a significant understanding of its pathogenesis, the precise causes of AIHA remain largely unclear and are thought to be multifactorial. In this paper, we presented a case of sickle cell anemia who developed severe AIHA that failed to maintain response to multiple treatment lines, including steroids, intravenous immunoglobulin, rituximab, and immune suppressive medications. However, a favorable response was achieved through the utilization of bortezomib. This report contributes to the expanding body of evidence regarding the efficacy of proteasome inhibitors in the management of severe and refractory autoimmune hemolysis in patients with sickle cell anemia.

## Introduction

Warm-type autoimmune hemolytic anemia (W-AIHA) is the major class of autoimmune hemolytic anemia (AIHA), comprising 60-70% of cases [1-3]. Its incidence is higher in females and increases with age, with the median age of presentation at 53 years. The most common etiology of W-AIHA is idiopathic, while others include cancers, medications, infections, and secondary to other autoimmune diseases [4]. The pathogenesis of AIHA is intricate and primarily involves several mechanisms: antibody production by the B-cell compartment, disruption of T-lymphocyte homeostasis, destruction of erythrocytes by the mononuclear phagocytic system, and activation of the complement systems. Also, the effectiveness of the marrow compensatory response played a crucial role in determining the severity of AIHA [1-4]. At body temperature, RBCs coated with IgG are destroyed by phagocytosis in the spleen, with or without complement involvement. The clinical symptoms vary from asymptomatic to life-threatening hemolysis. While the underlying cause of red cell destruction is immune-mediated, the risk of AIHA is increased in patients frequently exposed to donor red blood cells from frequent transfusions, such as patients with sickle cell anemia [5].

Managing cold-type AIHA and W-AIHA varies in the type and sequence of immunosuppressive agents used. Supportive measures such as blood transfusions, vitamins, erythropoiesis-stimulating agents, prophylactic anticoagulants, and antimicrobials are to be considered. Additionally, secondary AIHA requires treatment for the underlying cause, such as immunosuppressants for autoimmune diseases, chemotherapy or immunotherapy for lymphomas and other cancers, and immunoglobulins for primary immunodeficiency [1].

In a majority of cases, W-AIHA responds to first-line steroids; other treatments may include options such as immunosuppressants, rituximab, or splenectomy. However, in some cases, refractory or relapsed AIHA requires different treatment approaches that target the immune system, such as proteasome inhibitors and potent, selective, and reversible ubiquitin-proteasome system inhibitors that induce apoptosis of the cell [6]. Bortezomib, a proteasome inhibitor, was initially approved as an anticancer treatment for multiple myeloma and mantle cell lymphoma by the Food and Drug Administration in 2003. In addition to its anticancer properties, it is also thought to have an immunosuppressive effect by deactivating B- and T-lymphocytes, decreasing their migration and adhesion, and restricting MHC class I antigen presentation [6-8].

In this paper, we present a sickle cell disease patient who had a history of multiple blood transfusion reactions and developed W-AIHA that was severe and refractory to more than three standard lines of management and responded to bortezomib.

## Case presentation

A 28-year-old woman with sickle cell disease (HbSS) presented with severe signs and symptoms of anemia, including fatigue, shortness of breath, palpitations, and obvious signs of hemolytic anemia such as pallor, dark urine, jaundice, and hepatosplenomegaly. Her hemoglobin level was 4.6 g/dL, and a direct antiglobulin test (DAT) was positive for anti-IgG and anti-C3d, while IgM was negative (Tables 1-2). She was diagnosed with W-AIHA based on the definition provided by the First International Consensus Meeting in 2019, which describes W-AIHA as "lacking cold-associated symptoms with a DAT positive for strong positive IgG and C3d when a clinically significant cold-reactive antibody has been excluded."

**Table 1:** Patient’s lab values at presentation and after treatment WBC: white blood cells, Hct: hematocrit, MCV: mean cell volume, MCH: mean cell hemoglobin, MCHC: mean cell hemoglobin concentration, RDW-CV: red cell distribution width

Tests	Result at presentation	Results 8 months after treatment	Normal value
Hemoglobin	4.6 g\dl	8.42 g\dl	12-16 g\dl
WBC	25.8 x 10^9^/L	6.44 x 10^9^/L	3-11 x 10^9^/L
Platelet	118 x 10^9^/L	126.9 x 10^9^/L	155-450 x 10^9^/L
MCV	107.5 fl	92.1 fl	75-96 fl
MCHC	32.2 g/dl	31.5 g/dl	32-36 g/dl
Hct	14.3%	26.2%	38-52%
RDW-CV	33%	21.6%	11.5-14.5%
Bilirubin	3.7 mg/dl	1.85 mg/dl	0.1-1 mg/dl
LDH	1236.6 U/L	360.31 U/L	135-225 U/L

**Table 2:** Patient’s lab values on the day of presentation ANA: anti-nuclear autoantibodies, anti-ds DNA antibodies: anti-double-stranded DNA antibodies, LDH: lactate dehydrogenase, DAT: direct antiglobulin test, abdominal US: abdominal ultrasound

Other tests at presentation	Results
DAT (anti-IgG, anti-C3d)	+++
Reticulocyte count	High
Indirect Coombs test	Negative
Blood film	Features of hemolysis, including anisopoikilocytosis, polychromasia, nucleated RBCs, target cells, and irregularly contracted cells with spherocytes, as well as mildly reduced platelets
Virology screens	Negative Hep B, C, and HIV
ANA and anti-ds DNA antibodies	Negative
Abdominal US	Hepatosplenomegaly with the spleen measuring 19 cm and the liver measuring 18.9 cm. An adnexal cyst of dimensions 4*4*4 cm is reported as a functional ovarian cyst
Bone marrow biopsy	Cellular marrow with erythroid hyperplasia

Regarding her pregnancy history, she experienced recurrent typical sickle pains during the second and third trimesters, which were usually managed at home with simple analgesics such as paracetamol or Co-codamol. At 32 weeks of gestation, an abdominal ultrasound performed as part of her general pregnancy screening revealed mild splenomegaly, with a spleen size of 14 cm. This could be attributed to her pregnancy or partially to her sickle cell anemia. Although rare, splenomegaly has been reported in adults with homozygous sickle cell anemia and is associated with higher levels of HbF and a less severe disease course [7].

Her hemoglobin level was initially found to be 8 g/dL but paradoxically dropped to 3.5 g/dL following an RBC transfusion, accompanied by symptoms of dark urine and jaundice. The differential diagnoses included acute hyperhemolytic syndrome, delayed hemolytic transfusion reaction, or a hemolytic crisis related to her sickle cell anemia. She was initially treated with blood transfusions, but her hemoglobin levels did not improve. Subsequently, she was managed conservatively without further transfusions.

At the time of delivery at 39 weeks of gestation, her hemoglobin level was 6 g/dL. Tragically, her fetus died after birth, and neonatal alloimmune hemolytic anemia was suspected as the cause of neonatal mortality. One month postpartum, her symptoms had resolved, and her hemoglobin level had improved to 8.5 g/dL, with her overall condition being stable.

Aside from the above and occasional vaso-occlusive crises, her history of sickle cell disease has been unremarkable.

**Figure 1:**
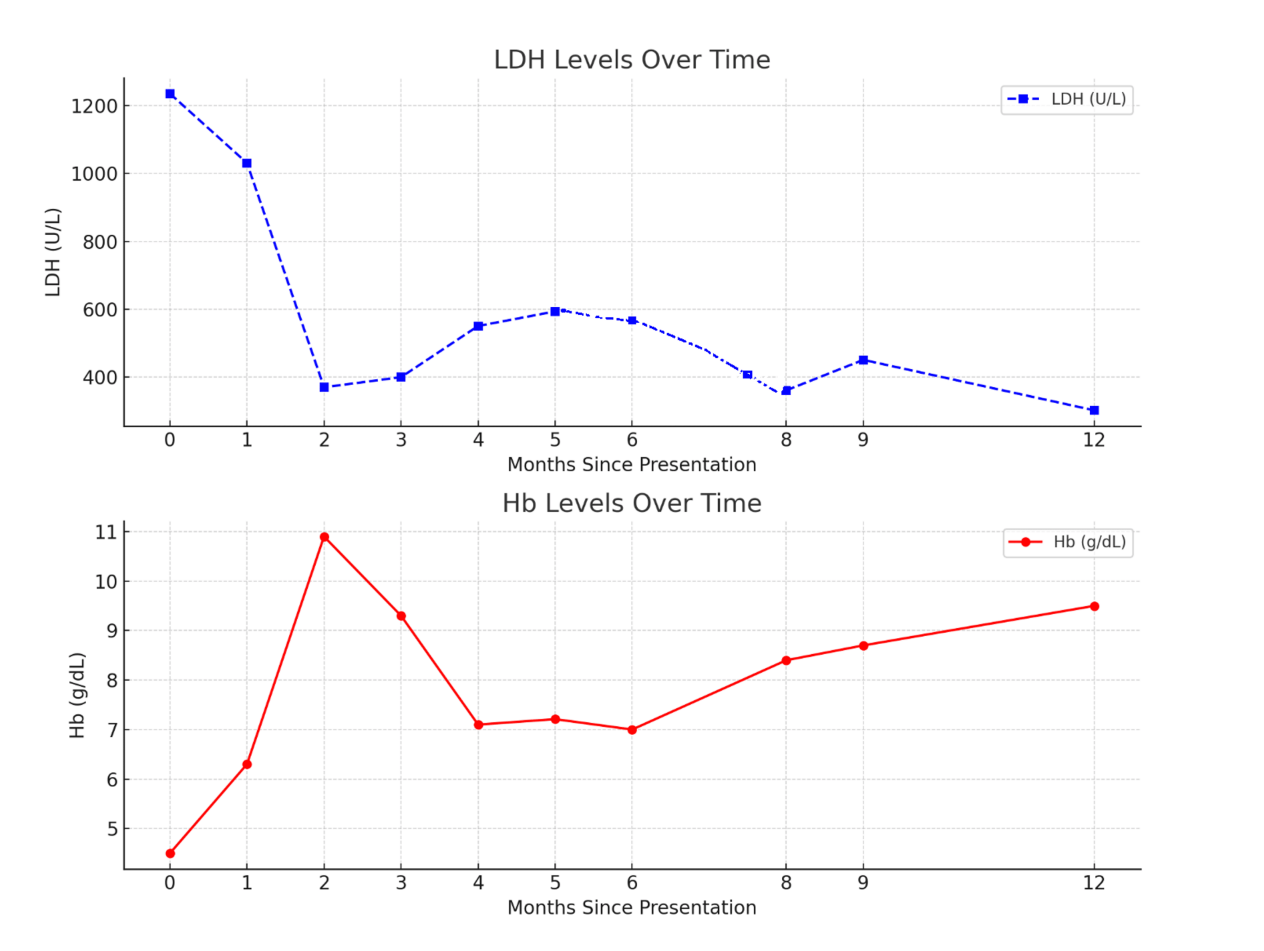
Top graph: LDH levels over time, indicating hemolysis trends. Bottom graph: Hb levels over time, showing the progression of anemia management LDH: lactate dehydrogenase, Hb: hemoglobin

Six weeks after the delivery of her second baby, she presented with a low hemoglobin level of 4.6 g/dL and a positive DAT. Prednisolone at 1 mg/kg/day was initiated (Table 3). A response was achieved, with hemoglobin levels increasing from 4.6 g/dL to 8.8 g/dL and subsequently to 10.5 g/dL within a few days. This improvement was accompanied by transfusion independence, although lactate dehydrogenase (LDH) levels remained high (Figure 1). According to the First International Consensus Meeting on AIHA in 2019, a response is defined as an increase in hemoglobin by >2 g/dL or normalization of hemoglobin without biochemical resolution of hemolysis, along with the absence of transfusions for the preceding seven days [8].

**Table 3:** Clinical progression and treatment response in a patient with hemolytic anemia IVIG: intravenous immunoglobulin, MMF: mycophenolate mofetil, SC: subcutaneous, Hb: hemoglobin, LDH: lactate dehydrogenase

Months since presentation	Treatment	Hb g/dl	LDH U/L	Bilirubin mg/dl
0	Start of prednisolone 1 mg/kg	4.5	1236	3.7
	Rituximab (375 mg/m²) was administered simultaneously with prednisolone every week for four weeks	8.8	1220	-
1	Tapering of steroids, but Hb dropped and IVIG given	6.3	1031	-
2	IVIG effect, continued tapering of steroids with azathioprine introduced as a steroid-sparing agent	10.9	369	1.85
3	MMF was introduced	9.3	399	-
4	Continuation of MMF, attempting to taper steroids further during this period	7.1	550	2.4
5	Continued MMF, and taper steroids	7.21	593	3
6	Start bortezomib 2 mg SC once weekly, steroid stopped	7	540	2
8	Bortezomib 2 mg SC once weekly	8.4	360	1.85
9	Bortezomib 2 mg SC once weekly	8.7	450	1.53
12	Bortezomib stopped	9.5	301	-

After achieving a response, tapering of the prednisolone dose was initiated after one month to minimize steroid-related side effects. The dose was gradually reduced by 5 mg every two weeks until reaching 10 mg. Total steroid treatment lasted three to five months, including the tapering phase. During this period, additional supplements were provided to ensure an adequate daily intake of vitamin D (600-800 IU) and calcium (1000-1200 mg). Rituximab (375 mg/m²) was administered concurrently with steroids once a week for four weeks.

However, whenever prednisolone was tapered below 20 mg, a drop in hemoglobin occurred, indicating steroid dependency. Steroid dependency is defined as the need to continue prednisolone at a dose >10 mg/day to maintain a response, as outlined in the "Diagnosis and Treatment of Autoimmune Hemolytic Anemia in Adults: Recommendations from the First International Consensus Meeting in 2019."

With steroid tapering, Hb dropped to 6.3 g/dL. Subsequently, IVIG (0.4 g/kg/day) was administered for five days, achieving a response that lasted for a few weeks. Eventually, third-line therapies such as azathioprine, cyclosporine, and mycophenolate were considered. The patient declined splenectomy and was instead started on azathioprine (100 mg OD) and mycophenolate mofetil (1000 mg BD). After more than five months of continuous therapy with prednisolone, rituximab, IVIG, and immunosuppressants, bortezomib was introduced (1.3 mg/m²) weekly for three months and then every two weeks, along with prophylactic acyclovir and vitamin B12. The total duration of bortezomib treatment lasted six months, after which she remained on folic acid only.

Bortezomib therapy enabled successful weaning from steroids and steroid-sparing agents. The Hb level remained between 8 g/dL and 9 g/dL for 10 months, consistent with the patient's baseline, without the need for transfusions. The patient demonstrated a good response to the proteasome inhibitor bortezomib, with resolution of anemic symptoms and no adverse effects or complications such as peripheral neuropathy.

## Discussion

This report sheds light on a challenging case of a patient with sickle cell disease and a chronic, severe, and refractory AIHA. Both sickle cell disease and AIHA result in hemolysis, increasing the burden on patients and complicating treatment for doctors, particularly when defining treatment response. As per recommendations from the first International consensus meeting in 2019, response to AIHA treatment is defined as an increase in Hb by more than 2 g/dL or normalization of Hb without the biochemical resolution of hemolysis, along with no transfusions in the last seven days of treatment, while a complete response is characterized by Hb normalization, no evidence of hemolysis (normal bilirubin, LDH, haptoglobin, and reticulocyte levels), and no transfusions [8]. However, in cases where the patient has a known history of sickle cell disease, differentiating immune hemolysis from sickle hemolysis biochemically to assess response is challenging, especially when defining a complete or incomplete response. The level of Hb and hemolysis markers compared to the baseline were used as a guide to assess response, in addition to transfusion status. A DAT was used to ascertain whether hemolysis possesses an immune origin due to IgM or IgG, which was strongly positive in this case and turned back to negative after two months of treatment. This distinction is significant because the treatment might differ. Positive DAT with IgG (with or without complement activation) and pan-agglutinin in the eluate sample aligns with the diagnosis of W-AIHA [8].

Published literature regarding combined sickle cell disease and AIHA is limited. Chaplin and Zarkowsky reviewed five patients who experienced combined sickle cell disease and AIHA within ten years. One patient had a positive DAT without verified autoimmune hemolysis. Severe hemolysis was observed in four patients, and their hemoglobin dropped rapidly, with severe anemia and reticulocytosis. For all patients, the hemolysis improved to steroids; mercaptopurine was given to one patient. DAT results returned to negative in all patients after treatment [9].

In this case, even immunosuppressive was not keeping the Hb near the baseline or stabilizing hemolysis markers; thus, proteasome inhibitors have been used to treat AIHA due to their ability to induce plasma cell apoptosis [10-12]. Bortezomib is the main proteasome inhibitor that has been used either on its own or accompanied by dexamethasone, rituximab, plasma exchange, and other drugs to manage AIHA, Evans syndrome, and ITP [7,13-17]. Corticosteroids result in a 70-80% response rate [5-9]. The relapsed/refractory W-AIHA with steroid dependency needs second-line treatment such as rituximab and splenectomy, or even third-line treatment such as immunosuppressive medications. Nevertheless, the efficacy of those treatments is insufficient and limited in some cases, accompanied by various adverse reactions [18-20].

In light of the diverse array of treatment modalities sought for AIHA, it is evident that numerous options exist, each with its own merits and limitations. However, amidst this diversity, the paramount consideration remains the patient's well-being and clinical response to treatment. While the literature suggests many therapeutic avenues, the best approach ultimately revolves around the one that optimally addresses the patient's specific condition, preferences, and clinical response.

Future research and clinical endeavors should strive not only to expand the arsenal of available treatments but also to personalize therapeutic strategies, ensuring that the chosen intervention aligns with the patient's needs, thereby fostering the highest quality of care in the management of AIHA.

## Conclusions

In cases of refractory W-AIHA, the potential efficacy of proteasome inhibitors, notably bortezomib, presents a promising avenue for therapeutic exploration. However, the current body of evidence necessitates further extensive research and clinical trials to comprehensively assess the safety, efficacy, and optimal application of these inhibitors in managing AIHA. For refractory cases of AIHA, continued investigation into the use of different treatments is warranted.
